# Tear ascorbic acid levels and the total antioxidant status in contact lens wearers: A pilot study

**DOI:** 10.4103/0301-4738.53054

**Published:** 2009

**Authors:** Sai Jyothi Aluru Venkata, Angayarkanni Narayanasamy, Vidhya Srinivasan, Geetha Krishnan Iyer, Ramakrishnan Sivaramakrishnan, Madhumathi Subramanian, Rajeshwari Mahadevan

**Affiliations:** Biochemistry Research Department, Sankara Nethralaya, Vision and Medical Research Foundation, Chennai, India; 1 Cornea Services, Sankara Nethralaya, Vision and Medical Research Foundation, Chennai, India; 2 Contact Lens Clinic, Sankara Nethralaya, Vision and Medical Research Foundation, Chennai, India

**Keywords:** Antioxidant, antioxidant capacity, ascorbic acid, contact lens wear, oxidative stress, tears

## Abstract

**Aims:**

The tear ascorbate owing to its high concentration, functions as an effective antioxidant against the oxidative damage of cornea. Contact lens wearers (CLW) are prone to oxidative stress due to the lens-induced hypoxic conditions. A pilot study was done to compare the tear ascorbic acid level and the total antioxidant capacity give as in normal and CLW.

**Materials and Methods:**

In this study 21 CLW (Mean age 23 ± 3 years; M-2, F-19), who were daily wear users, with duration of wear not more than four years, along with age-matched 28 controls (Mean age 28 ± 3; M-15, F-13) were recruited in the study for collection of reflex tears using Schirmer's strip. Ascorbic acid in tears was determined using high-performance liquid chromatography (HPLC), total antioxidant capacity (TAC) and total protein assay by spectrophotometric analysis.

**Results:**

CLW showed no significant change in the tear ascorbic acid levels (0.4 ± 0.26 mM) compared to the control subjects (0.61 ± 0.59 mM). The amount of ascorbic acid in tears did not correlate with the TAC or the total protein of the tears. The mean TAC in CLW was 0.69 ± 0.16 mM, with a total protein of 1.35 ± 0.46 mg/ml while in controls it was 0.7 ± 0.18 mM and 1.21 ± 0.47 mg/ml respectively.

**Conclusions:**

Soft contact lens wear did not show any significant change in tear ascorbic acid, TAC and total protein levels compared to controls.

The cornea is particularly at risk from oxidative damage, owing to direct light exposure and environmental insults such as air pollutants and ultraviolet (UV) radiation.[1] The free radical and reactive oxygen species (ROS) generation can trigger corneal damage owing to potential modifications of proteins, lipids, and DNA.[2] Tear film alterations in various conditions including contact lens (CL) wear are complex and involve not only tear quantity but also tear quality.[3,4]

Of the many antioxidants in the tear film such as cysteine, glutathione, urate, and tyrosine,[5] ascorbate (vitamin C) *with its high* reducing power and water-soluble property,[6] makes it a major and an effective antioxidant. Ocular tissues and fluids contain particularly high levels of ascorbate, reflecting high demand for antioxidant protection.[7] Ascorbate is also reported to enhance wound healing and control inflammatory processes in the cornea[8] and therefore has been used therapeutically for many corneal disorders, including alkali burns[9] and inflammation after excimer laser corneal surgery.[10] It has been reported that tears may provide a continuous source of ascorbate for corneal epithelium, serving a dual function as antioxidant defense and acting as the vector for ascorbate uptake by the cornea.[11] Choy *et al*. suggest that the ascorbate can leak from the corneal epithelial cells into the tear film, if epithelial cell damage of the cornea is sustained. Thus the tear ascorbate levels can be related to the corneal health status.[11]

The measurement of total antioxidant activity of tear sample is a composite measurement of all the scavenging antioxidants within the sample and may provide insight into the overall prevalent antioxidant balance.[12]

In addition to antioxidants, the tear film also contains proteins such as immunoglobulin A, lipocalin, and other proteins which might protect the cornea from oxidative damage.[13,14]

Contact lenses (CL) are increasing in popularity as new materials and designs render them easier to fit and are more comfortable to wear.[10] CL have a wide variety of effects on the cornea attributable to their mechanical effect and to their tendency to impair oxygen delivery to the cornea.[15] Contact lens wearer (CLW) also has potential complications like CL-induced acute red eye (CLARE), CL peripheral ulcers (CLPU), infiltrative keratitis and microbial keratitis.[16]

Since the epithelium derives virtually all of its oxygen from the atmosphere, CLs have the capability of interrupting that supply.[17] Tear antioxidants (mainly ascorbic acid) might play an important role in the corneal health status of CLW. There has been no study so far, to see if there is an alteration in tear ascorbic acid levels in CLW. The aim of the pilot study was to compare the levels of ascorbic acid, total antioxidant capacity (TAC) and total protein in the tears between soft CLW versus and non-CLW.

## Materials and Methods

This pilot study was approved by the Ethics committee of the Institutional Research Board. The CLW in the study were users for not less than two months and not more than four years. The CLW used only soft daily wear hydrogel contact lenses and were recruited with their informed consent. The wear schedule of CL was 12-16 h. The soft CLs used were from either Bausch and Lomb or Johnson and Johnson and the lens materials varied (hexa ethyl methyl acrylate-HEMA, Etafilcon A, Hilafilco B, Aphafilcon B) with Dk values ranging from 9 to 40. Sixty-two per cent of the study subjects used HEMA with Dk value of 9. The rest included all other kinds.

The CLW recruited in the study were volunteers from the Institute who were employees and students, and had a mean age of 23 ± 3 years (range: 19-32 years) with 19 females and two males (total of 21 cases). The age-matched controls had a mean age of 28 ± 3 years (range: 20-32 years) with 15 females and 13 males (total of 28 subjects). Subjects with no history of CL wear or ocular pathology were recruited as controls. A questionnaire was used to assess the control group and the CLW to rule out history of systemic and ocular pathologies. Those subjects who had taken any vitamin supplements, oral medication or who had systemic or ocular pathology were excluded from the study.

Reflex tears were collected using the Schirmer strips (Contacare, Baroda, India) by placing the strip in the lower cul-de-sac and tears were allowed to diffuse into the strip for 5 min. Only one eye was used for all the sample collection and the subjects were allowed to freely blink while collecting. The tear specimens collected using the Schirmer's strip were placed in polyethylene vials to prevent evaporation of samples and then immediately processed for extraction.

It has been demonstrated that weighing the wetted paper strips before and after tear collection gives an accurate determination of the collected tear volume.[18] After weighing the Schirmer strips before and after tear collection, they were subjected to extraction. For extraction, the Schirmer's strips were mixed with 200 µl of 2mM homocysteine (Sigma, St. Louis, USA) for 30 sec, incubated at 4°C for 10 min, mixed on a vortex mixer for 1 min and then centrifuged at 3000 rpm for 3 min. The supernatant was immediately used for ascorbic acid assay as per the standard protocol for vitamin C extraction from Schirmer's strips and estimation in tear samples by high-performance liquid chromatography (HPLC) as given by Howard *et al*.[19] Twenty microlitres of supernatant of the Schirmer's strip extract was injected directly into the HPLC column within 30 min of extraction. The rest of the supernatant was then aliquoted for TAC and total protein assay and stored at −20°C until analyzed.

### Standard

A standard calibration curve was made using stock solution of ascorbic acid (Sigma St. Louis, USA) diluted in aqueous homocysteine (2mM), a known antioxidant, for concentrations ranging from 100-500ng in a volume of 20 µl.

### HPLC analysis of ascorbic acid

For the analysis of ascorbic acid, Shimadzu HPLC with UV detector system was used. HPLC column conditions for the assay include the use of C18 column (150 × 4.6mm, 5mm diameter column) with isocratic elution using mobile phase of 0.1% orthophosphoric acid: Methanol (1:1 ratio), pH - 2.8, with a flow rate of 1.0 ml/min, at a column temperature of 40°C. The detection was at 245nm by the UV detector. The HPLC grade solvents (Merck) were degassed using ultrasonicator before use. The run time per analysis of sample was 10 min and the analysis was done according to the protocol of Howard *et al*.[19] The ascorbic acid was eluted at 1.5^th^ min, based on the above column conditions. Calibration was done using 100–500ng of standard ascorbic acid. The tear samples also showed the R_t_ (retention time) of ascorbic acid at the same 1.5^th^ min. This was also checked and confirmed by spiking the sample with known concentration of standard ascorbic acid.

### Recovery of the ascorbic acid from the Schirmer's strip with and without tear

100, 200, 300ng of standard ascorbic acid was added to the Schirmer strip with and without tear followed by extraction of the ascorbic acid, followed by HPLC analysis.

### TAC assay

This involves the formation of iron-ethylene diamine tetra acetic acid (Fe-EDTA) complex that reacts with hydrogen peroxide by a Fenton type reaction, leading to the formation of hydroxyl radicals (^•^OH), a reactive oxygen species that degrades benzoate resulting in the thiobarbituric acid reactive substances (TBARS) formation. Antioxidants present in the tear elute from the Schirmer's strip cause suppression of the TBARS. Amount of TBARS is read spectrophotometrically at 535 nm.[20] The fall in the absorbance gives TBARS suppression and gives the measurement of TAC which is expressed in terms of TBARS as mM (mmoles/liter)

### Protein assay

The total protein in the Schirmer's strip elute was determined by *Lowry* method.[21]

For statistical analysis, the Student's paired *t-test* was performed to determine differences between samples. For correlation studies, Pearson's correlation co-efficient was used (SPSS software, version 14.0).

## Results

Table 1 shows the details of the CLW, with duration, type of wear and the amount of tears adsorbed onto the Schirmer's strip. The amount of tears adsorbed onto the Schirmer's strip for the control (non-CLW) subjects was 30 ± 8 mm with a range of 9-35mm and for the CLW it was 29 ± 8 mm with a range of 11-35mm. The tear secretion in both the groups was found to be comparable and was not significantly different (*P* = 0.84).

**Table 1:** Details of the contact lens wearers in the study

Age/sex	*Duration of CL wear	Schirmer (mm)
22/F	3 years	11
23/F	2 years	35
32/F	3 years	12
25/F	2 months	29
22/F	1 1/2 years	24
23/F	4 years	23
21/F	2 years	34
24/F	4 years	35
19/F	4 months	35
22/F	11 months	35
24/F	3 years	32
20/F	3 years	28
22/F	1 year	35
19/F	1 year	35
20/M	6 months	35
24/F	3 years	30
26/M	6 months	30
24/F	3 years	11
22/F	5 months	35
21 /F	4 years	12
22/F	1 year	29

* Type of lens used - soft daily wear hydrogel contact lenses, CL: contact lens

The ascorbic acid in the standard and the tear specimen showed a retention time (R_t_) of 1.5 min by HPLC. The recovery of the ascorbic acid after spiking known concentrations of ascorbic acid on the Schirmer's strip followed by extraction was found to be 97% for standard alone and 98% with the tear sample spiked with the standard.

**Figure 1:**
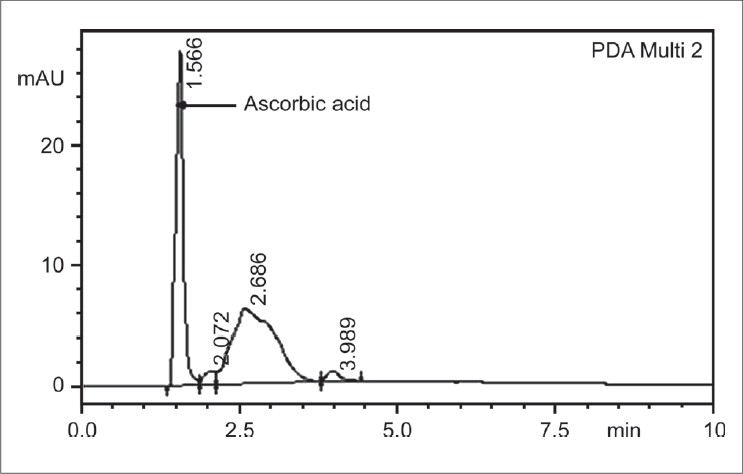
HPLC profile of standard ascorbic acid

**Figure 2:**
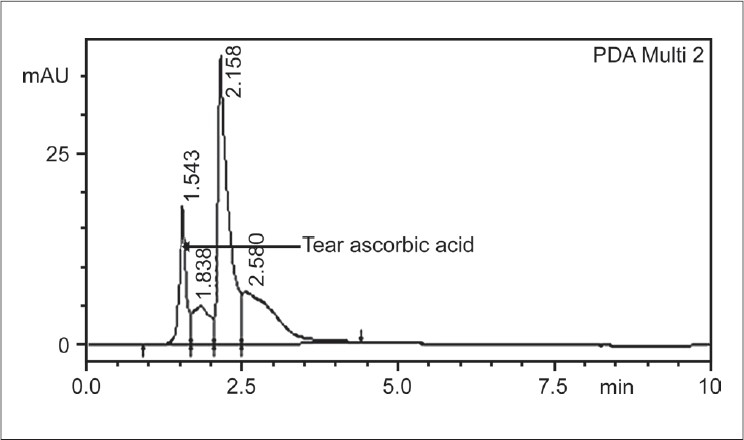
HPLC profile of tear ascorbic acid

Fig. 1 shows the standard ascorbic acid peak with R_t_ of 1.5^th^ min at the given column conditions.Fig. 2 shows the tear ascorbic acid eluted at the same R_t_ i.e. 1.5^th^ min. Ascorbic acid level in the control non-CLW subjects was 0.61 ± 0.59mM (mmoles/liter) with a range of 0.15-2.57mM (mmoles/liter) The mean ascorbic acid level in CLW was 0.4 ± 0.26mM, with a range of 0.05-1.1 mM. The ascorbate levels in CLW was not statistically significantly different from that of the control (*P* = 0.14).

The TAC in the tear sample of controls was 0.7 ± 0.18mM, with a range of 0.41-1.03mM, and in the CLW it was 0.69 ± 0.16 mM, with a range of 0.46-1.02mM [Table 2]. There was no significant correlation between the TAC and ascorbic acid levels in both controls (r = 0.38 and *P* = 0.14) and CLW (r = 0.02, *P* = 0.23).

**Table 2:** Ascorbic acid, total protein and total antioxidant capacity in contact lens wearers and controls

Parameters	Ascorbic acid (mM)	Total protein (mg/ml)	TAC (mM)
Controls			
Mean	0.61 ± 0.59	1.21 ± 0.47	0.7 ± 0.18
Median	0.39	1.32	0.665
Range	0.15-2.57	0.27-1.95	0.41-1.03
Contact lens wearers			
Mean	0.4 ± 0.26 *(P = 0.14)*	1.35 ± 0.46	0.69 ± 0.1 6
Median	0.34	1.32	0.66
Range	0.05-1.1	0.72-2.54	0.46-1.02

TAC - Total antioxidant capacity

Similarly, there was no difference in the total protein concentration of tear samples between the two groups. The total protein concentration of controls was 1.21 ± 0.47 mg/ml with a range of 0.27-1.95mg/ml), and in CLW was 1.35 ± 0.46mg/ml, with a range of 0.7-2.54mg/ml [Table 2].

## Discussion

The antioxidant status of tear fluid is of interest because tears constitute the first barrier protecting the vulnerable cornea.[5] Though there are different tear antioxidants, ascorbic acid and urate contribute to 50% of the total antioxidant capacity in tears.[22] Vitamin C supplementation is reported to increase the tear ascorbate levels apart from that in plasma.[23] Very high concentration of ascorbic acid in the corneal epithelium results from the ‘sodium-dependent vitamin C transporter 2’ that is present in both the ciliary epithelium and the corneal epithelium.[24] Ascorbic acid is transported from the plasma to the aqueous humor and then from the aqueous humor into the corneal epithelium. According to Ringvold's hypothesis, ascorbic acid shows an excellent absorption of UV radiation between 280 and 310 nm and it has an absorption curve that roughly matches the absorption curves of protein and nucleic acids, thereby acting as a physiological ‘sunscreen’.[25] The concentration of the ascorbate in the corneal epithelium is the highest amongst all tissues with a cytosolic concentration of 11mM.[25] Therefore the tear ascorbate is of physiological importance.

Extended wear CLs from highly oxygen-permeable soft materials are available and are required for normal functioning of the cornea although the most commonly used CLs for extended wear are of lower oxygen permeability. Since hypoxia can induce the production of antioxidants to protect the cornea from the oxidative stress, we estimated the tear ascorbic acid levels and the TAC in the reflex tears of CLW using soft lens within four years of duration.

This study demonstrates no significant difference in the tear ascorbic acid levels between CLW and non-CLW. However, the limitations of the study include the smaller sample size of the study with variation in factors such as duration of the CL wear, type of CL material and degree of oxygen permeability of the soft CLs.

Similarly, the TAC of the tears in these two groups was comparable, with no statistical difference. Choy *et al.,*[22] showed that tear TAC in non-CLW was 0.46 ± 0.16 mM by Ferric reducing antioxidant activity and ascorbic acid concentration (FRASC)method and this is in agreement with the current study of 0.7 ± 0.18mM as determined by the method of Koracevic *et al*.[20]

The concentration of total proteins in tears in these two groups in this study was again not statistically significant. Stapleton *et al.,* reported that the total protein content of tears was increased during overnight CL use compared to the 12 h of daily CL wear.[26] Overnight CL use causes increased protein due to reduced tear turnover during eye closure, as active tear flow is suppressed with cessation of the blink reflex.[26]

There was no alteration in the tear production in CLW compared to the non-CLW which is perhaps indicative of normal tear production in these two groups. Reduced Schirmer values have been reported in case of CLW in dry eye.[26] In the current study no confirmatory tests or clinical signs and symptoms were looked for, to strongly comment on the corneal health status apart from Schirmers', and questionnaire.

In conclusion, this pilot study reveals that in the absence of any obvious ocular complications, there are no significant changes in the levels of ascorbic acid, total protein and TAC in the tear samples of CLW using soft hydrogel CLs and wearing for not more than four years, compared to non-CLW. Further study with a larger sample size in CLW associated with corneal pathology or inflammation can yield more information on the corneal health status with respect to ascorbic acid.
